# Use of Virtual Assistant Devices to Improve Depression in Cognitively-Impaired Homebound Older Adults

**DOI:** 10.1093/geroni/igaf122.1994

**Published:** 2025-12-31

**Authors:** Jessica Lee, Jason Burnett, Prisha Patel, Matthew Nunez

**Affiliations:** The University of Texas Health Science Center at Houston, Houston, Texas, United States; UTHealth Houston, Houston, Texas, United States; UTHealth Houston, Houston, Texas, United States; UTHealth Houston, Houston, Texas, United States

## Abstract

Technology is progressively playing a larger role in individual health and wellness, with increasing use seen in older adults. However, older adults can face uncertainty and inadequate understanding of the features of such devices. Homebound older adults, in particular, often have cognitive or physical impairments that may heighten barriers to technology use. Therefore, we wanted to assess the feasibility and acceptance of virtual assistant device use in cognitively impaired homebound older adults. We partnered with our local Meals on Wheels to evaluate whether the Amazon Alexa Show 8 (AES8) could be appropriately utilized and potentially improve depression in clients aged 60 and older. Participants went through three phases of the study: 6 weeks of meal deliveries alone (control), followed by 6 weeks of meals+AES8 basic usage, and lastly 6 weeks of meals+AES8 advanced usage. Participants had improvements in technology acceptance and associations with improvements in depression and memory by the end of the study, despite no specific health programming. Participants anecdotally enjoyed being able to play music, set reminders, and access spiritual content on the devices. These results have led to a subsequent pilot study evaluating the feasibility of remote installation of the AES8 with evaluations of loneliness and social isolation. Preliminary data indicates that cognitively intact homebound older adults are successfully able to install the devices with telephone assistance. Being able to reach larger numbers of homebound older adults with these virtual assistant devices, may mean increased potential to improve the mental health of high-needs homebound older adults.

